# Gene, pathway and network frameworks to identify epistatic interactions of single nucleotide polymorphisms derived from GWAS data

**DOI:** 10.1186/1752-0509-6-S3-S15

**Published:** 2012-12-17

**Authors:** Yu Liu, Sean Maxwell, Tao Feng, Xiaofeng Zhu, Robert C Elston, Mehmet Koyutürk, Mark R Chance

**Affiliations:** 1 Center for Proteomics and Bioinformatics, Case Western Reserve University, Cleveland, OH, USA; 2 Department of Epidemiology and Biostatistics, Case Western Reserve University, Cleveland, OH, USA; 3 Department of Electrical Engineering & Computer Science, Case Western Reserve University, Cleveland, OH, USA; 4 Department of Genetics & Genome Sciences, Case Western Reserve University, Cleveland, OH, USA; 5 NeoProteomics, Inc., Cleveland, OH, USA

## Abstract

**Background:**

Interactions among genomic loci (also known as epistasis) have been suggested as one of the potential sources of missing heritability in single locus analysis of genome-wide association studies (GWAS). The computational burden of searching for interactions is compounded by the extremely low threshold for identifying significant p-values due to multiple hypothesis testing corrections. Utilizing prior biological knowledge to restrict the set of candidate SNP pairs to be tested can alleviate this problem, but systematic studies that investigate the relative merits of integrating different biological frameworks and GWAS data have not been conducted.

**Results:**

We developed four biologically based frameworks to identify pairwise interactions among candidate SNP pairs as follows: (1) for each human protein-coding gene, a set of SNPs associated with that gene was constructed providing a gene-based interaction model, (2) for each known biological pathway, a set of SNPs associated with the genes in the pathway was constructed providing a pathway-based interaction model, (3) a set of SNPs associated with genes in a disease-related subnetwork provides a network-based interaction model, and (4) a framework is based on the function of SNPs. The last approach uses expression SNPs (eSNPs or eQTLs), which are SNPs or loci that have defined effects on the abundance of transcripts of other genes. We constructed pairs of eSNPs and SNPs located in the target genes whose expression is regulated by eSNPs. For all four frameworks the SNP sets were exhaustively tested for pairwise interactions within the sets using a traditional logistic regression model after excluding genes that were previously identified to associate with the trait. Using previously published GWAS data for type 2 diabetes (T2D) and the biologically based pair-wise interaction modeling, we identify twelve genes not seen in the previous single locus analysis.

**Conclusion:**

We present four approaches to detect interactions associated with complex diseases. The results show our approaches outperform the traditional single locus approaches in detecting genes that previously did not reach significance; the results also provide novel drug targets and biomarkers relevant to the underlying mechanisms of disease.

## Background

The typical analytic framework for the genome-wide association studies (GWAS) of complex diseases or traits considers the additive contribution of common variants (usually SNPs) to genetic risk one at a time, based on an assumption of an underlying simplified genetic architecture [1-4]. In the last few years, more than 400 GWAS have identified an unprecedented number of candidate disease-associated DNA sites (> 1,200) [5], many of them already well-known for their disease association, while many others have also been replicated and confirmed by follow-on studies [5]. However, for a given disease/trait, the genetic variation explained by GWAS is significantly less than the estimated heritability [6]. In addition, although the individual SNP contributions are considered independent with respect to additive heritability when Linkage Disequilibrium (LD) is taken into account, their independence in contributing to a complex disease is by no means assured. The potential for gene-gene interaction has been proposed to be one of possible reasons for the so-called "missing heritability" of GWAS, along with other possible factors such as rare variants and environmental factors. In addition, biological interactions may be very important in contributing to overall disease outcomes [6,7]. In this paper we come to the problem of GWAS analysis using an alternative assumption, not one of the independent action of SNPs or genes but rather one that assumes that they may indeed interact in causing complex disease. This is a well-known idea and when genes function primarily through a complex mechanism that involves multiple genes, the joint effect (behavior) of those genes' variants is referred to as a gene-gene interaction (or epistasis) [8-10], though biological interaction and statistical interaction are often confused [11]. The contributions of gene-gene interaction to the risk of diseases have been well documented, e.g., in the case of breast cancer and coronary heart disease [8,12].

One of the challenges in detecting gene-gene interactions is the computational burden due to the size of the search space for exhaustive pairwise testing [10]. Consequently, most methods employ either a heuristic or a two-step (screen-testing) approach, which may miss some true interactions [13]. However, some recent methods are reported to be efficient enough for an exhaustive search of GWAS data in a relatively short time [14,15]. An alternative approach to probing the effects of multiple genes is gene set enrichment analysis (such as GSEA-SNP and others [16,17]), which can serve to identify pathways that are implicated in disease. However, this approach considers all the genes in the pathway as equal, and cannot reveal the discrete structure of potential relationships of mechanistic interest. Another challenge in multi-SNP analysis is that the statistical significance threshold (p-value) has to be extremely low due to multiple hypothesis-testing corrections; typically, the p-value has to be smaller than 10^-13 ^to be significant for an exhaustive search in GWAS data and, very few interactions can pass such stringent thresholds [14]. On the other hand, one way to tackle this challenge is to use biological knowledge to narrow the statistical search space [18] and this idea dominates the approach taken here.

The progress of biomedical research over the last several decades has resulted in the accumulation of vast amounts of biological information stored in public databases, including gene/genome annotations, pathway, and network information. The analysis of GWAS data can benefit from the application of such rich resources. Various biological frameworks have been integrated with GWAS to detect biomarkers or pathways associated with complex diseases [19-23]. Recently, a number of approaches have been suggested to guide the search for gene-gene interaction based on the use of prior biological knowledge [18,24-26]. These approaches either focused on a single pathway or were based on interaction databases, and thus reduced the search space dramatically. However, because the databases are far from complete, using these alternative approaches alone would not identify novel interactions that are absent in the current interaction databases.

A recent study described a method to search for what could be interactions within the local SNP "neighborhood", but was proposed rather as a simple way to detect associated haplotypes, and demonstrated that this approach is efficient in detecting new disease associations [27]. By applying logistic regression to test adjacent SNPs pairs, six pairs of SNPs were found to significantly associate with coronary artery disease (CAD), and one pair locates in a known major CAD-associated region (9p21) [27]. Encouraged by this success, here we propose new and broader approaches to integrate the latest available gene annotation, pathway, and network information, along with the functionality of eSNPs, to detect gene-gene interactions associated with complex diseases. Briefly, the statistical interactions among SNPs involved in the same genes and pathways were exhaustively tested; and we also constructed a disease related subnetwork based on prior knowledge, and the interactions among genes in the subnetwork were also exhaustively searched.

Many searches for interactions based on biological information and annotation have focused on the SNPs located in coding gene regions. However, the vast majority of SNPs are located in intergenic regions, many of them are likely to have unannotated biological functions, and their potential interactions have been neglected in previous studies. Recent studies showed that many intergenic genomic loci can modulate gene expression by both *cis *and/or *trans *mechanisms, and the loci identified in this manner are referred to as expression quantitative trait loci, or eQTLs [28,29]. Highly significant associations between SNPs located in eQTLs and gene expression in various tissues have been established (such SNPs are referred to as expression SNPs, or eSNPs) [30-32]. Furthermore, one recent study has demonstrated that SNPs associated with complex traits are significantly more likely to be eSNPs [33]. However, the potential interactions related to eSNPs have not been studied and reported. In this study, we propose general methods to search for the interactions between eSNPs and SNPs located in a gene whose expression is affected by the eSNPs.

We have applied the approach of using several biological frameworks (gene, pathway, network, and eSNP) to reduce the relevant search space and make exhaustive pairwise searches tractable. Using these frameworks, which can be generalized to all complex diseases, we probe for pairwise SNP statistical interactions to provide novel candidate genes associated with type 2 diabetes (T2D) using the Wellcome Trust Case Control Consortium (WTCCC) data [1]. The results illustrate that our approach can detect multiple, new disease associations for complex diseases, which can point to novel biomarker and drug targets that illuminate the molecular mechanisms underlying the diseases.

## Methods and materials

### Data set

We obtained permission to access the WTCCC dataset for T2D from the consortium websites (https://www.wtccc.org.uk/info/access_to_data_samples.shtml, [1]). The detailed description of the study samples can be found in the original report. In brief, the data set has a pool of 3,004 controls (which consist of a 1958 Birth Cohort (1,504 individuals) and a recently recruited UK Blood Service sample (1,500 individuals)), and 1,999 T2D affected individuals. The majority of subjects were of European ancestry. Samples from the individuals were genotyped using Affymetrix GeneChip 500K arrays. The genotypes estimated with the algorithm CHIAMO were used in this study. The following exclusion criteria were used for quality control: (i) Hardy-Weinberg Equilibrium exact test P value < 5 × 10^-7 ^in controls; (ii) allele frequency difference test based on 1 degree of freedom (df) trend test P value < 5 × 10^-7 ^between the two control groups; (iii) minor allele frequencies < 1%. We were most interested to see if a focus on interactions could promote SNPs that were non-significant to significant status. After filtering and taking out the SNPs in genes found by single SNP analysis, the number of SNPs analyzed decreased from 500,568 to 418,097.

### Construction of SNP pairs for interaction testing

**Figure 1:**
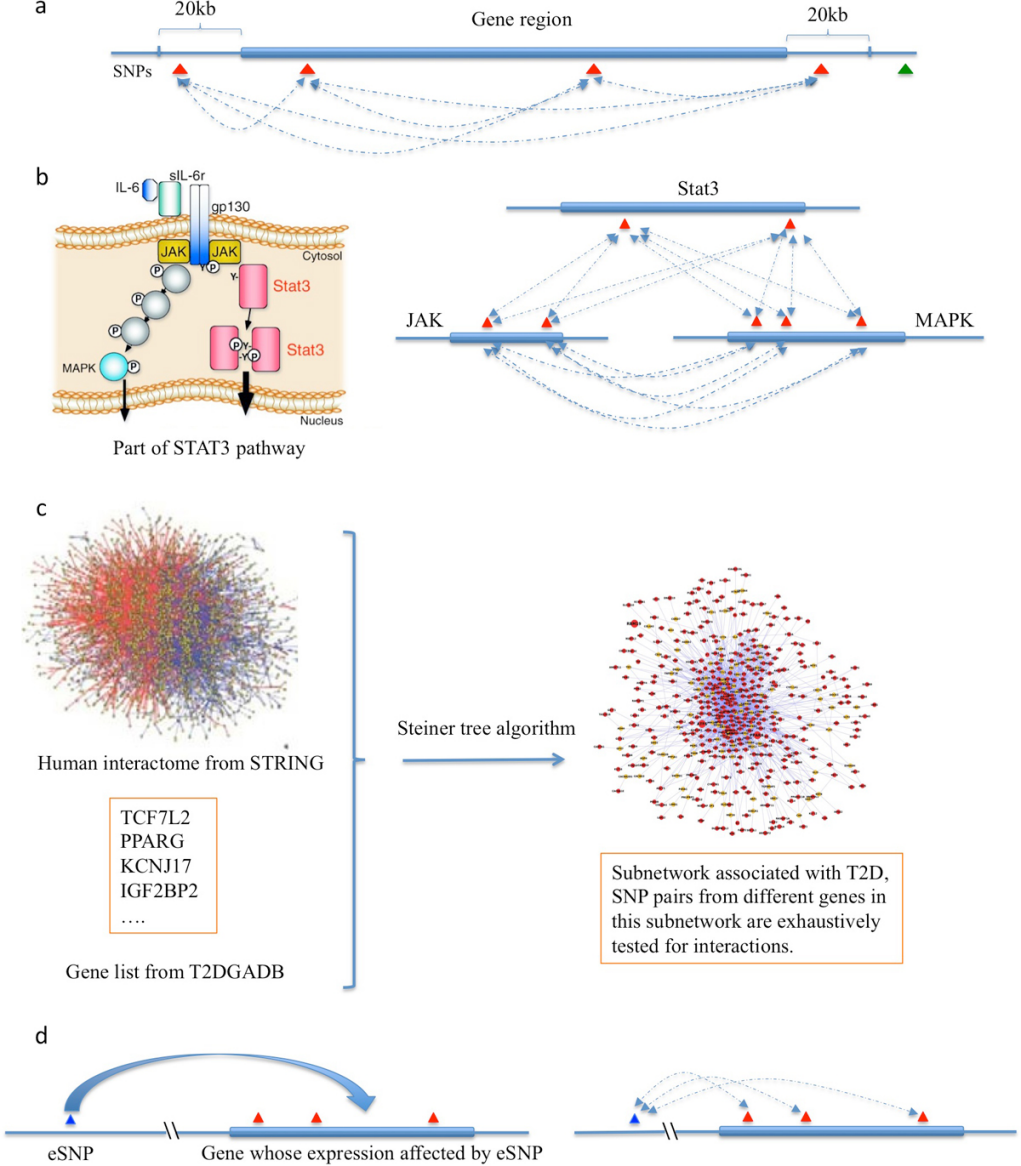
**Procedure to detect interactions among selected SNPs**. a) gene-based method: interaction among SNPs located in the same gene region (including 20 kb up and down stream of the gene) are exhaustively tested. Note that the previous method based on adjacent SNPs [27] can be considered a special case of this method; b) pathway-based method, part of STAT3 pathway are used to illustrate this method, interactions among SNPs located in different genes in the same pathway are tested; c) network based method: first, a disease-associated subnetwork is generated, then interactions among SNPs positioned in different genes in the subnetwork are tested; d) interactions between eSNPs and SNPs located in the genes whose expression are regulated by the eSNP are tested. Red triangles represent SNPs. Dashed lines represent potential interactions.

SNP pairs were generated based on gene annotation, pathway, and network knowledge, respectively. Briefly, a set of all SNP pairs in the same gene was constructed based on their genomic coordinates. Similarly, for each pathway, a set of all SNP pairs was created comprising SNPs located in different genes involved in the pathway. In the case of the network-based approach, a disease-associated network was first extracted from a human interactome database; then, SNP pairs with each member of the pair positioned in a different gene of the network were generated. The overall procedure is illustrated in Figure 1.

#### SNPs located in the same gene

We downloaded the genomic coordinates of 18,657 genes from the plink website (http://pngu.mgh.harvard.edu/~purcell/plink/res.shtml, [34]), which were generated from the UCSC table browser for all RefSeq genes on July 24th 2008. The coordinates of SNPs from the WTCCC dataset were used to map them to these genes. Because of the potential that regulatory elements could be located in the annotated gene neighborhood, 20 kb of sequence up and down stream of a gene was also considered as part of the gene. This may associate more than one gene with a single SNP. Gene assignments using this approach were used for the pathway, network, and eSNP analyses below.

To reduce the computational burden, only genes with less than 100 SNPs were analyzed. This constraint could be relaxed in future studies by considering the Linkage Disequilibrium (LD) structure, where SNPs located in the same LD blocks are highly correlated, and the genotyping information becomes redundant. The final analyzed set has 15,953 genes, which include 205,402 SNPs in total. For each of the 15,953 genes, SNP pairs were generated exhaustively based on SNPs located in the same gene, and tested for interactions. In total, more than 2.7 × 10^6 ^SNP pairs were tested. To correct for the multiple hypothesis-testing problem, a Bonferroni correction was used with a p-value cutoff of 1.85 × 10^-8 ^for a significance level of 5%.

#### SNP pairs in the same pathway

Canonical pathway data were downloaded from the Molecular Signatures Database (http://www.broadinstitute.org/gsea/msigdb/index.jsp, c2.cp.v3.0). The initial data contain 880 canonical pathways. Some pathways have very general functions, and contain large numbers of genes, e.g., the gene expression pathway from the Reactome has 425 genes, and the pathways in cancer from KEGG have 328 genes. To focus on pathways with more specific functions and to increase computational efficiency, only pathways with less than 50 genes were analyzed in this study. The final set has 655 pathways, and 1.9 × 10^5 ^SNPs in total. For each pathway, the interactions among SNPs located in different genes were tested, which led to 2.7 × 10^7 ^tests in total.

#### SNPs in a subnetwork associated with T2D

The subnetwork associated with T2D was constructed in three steps: 1) first, a human protein-protein interactome was downloaded from a public database; 2) then, genes associated with T2D (T2D genes) were also obtained from a database curated by literature mining; 3) finally, the T2D genes were used as seeds to extract a subnetwork from the interactome by applying the Steiner tree algorithm. The details for each of these steps are as follows.

The human interactome was downloaded from the STRING database maintained by EMBL (http://string-db.org/, [35]). Note that STRING contains known and predicted protein/gene interactions, which include direct (physical) and indirect (functional, such as mRNA co-expression) associations. They are derived mainly from four sources: high-throughput experiments for interaction detection (yeast two-hybrid, affinity purification followed by mass spectrometry, whole genome expression, literature mining, and genomic context). Based on the strength of evidence for each interaction, a score is assigned to reflect the confidence level. Those interactions with a score more than 0.80 were extracted to generate the human interactome, which contains 10,571 genes and 286,876 interactions.

Genes associated with T2D (T2D genes) were downloaded from a public database (T2DGADB, http://t2db.khu.ac.kr:8080/, [36]), derived from 701 publications of T2D association studies. 446 T2D genes showed disease association in from one to 49 publications. T2D genes (seed genes) were mapped to the interactome, and a T2D related subnetwork was constructed by adding new genes to connect T2D genes using a Steiner tree algorithm. Details of this algorithm can be found in the original paper and its applications [37-39]. Briefly, as a first step, T2D genes absent from the interactome are removed, then the algorithm adds other genes to connect the remaining genes, finally, the network is simplified based on the criterion of the shortest paths between seed genes. The final subnetwork has 453 genes and 2374 interactions, and 354 genes are initial T2D genes (seed genes) while 99 genes (nodes) are added to optimize the connectivity.

The SNPs located in the 453 genes of the subnetwork were collected, and the SNP pairs were exhaustively generated from all SNPs. SNP pairs from the same genes were removed. The final SNP pairs were tested for interactions, which results in 4.7 × 10^7 ^tests.

### Interaction between eSNPs and genes

To detect the interactions involved in SNPs located in intergenic regions, we analyzed the interactions of SNP pairs between eSNPs and SNPs positioned in the genes whose expressions are affected by the eSNPs.

The association data between eSNPs and genes was downloaded from a previous study (http://www.sph.umich.edu/csg/liang/asthma/, [31]) and public database (http://www.scandb.org, [40]). The p-value cutoff (< 10^-5^) was used to filter out the unreliable associations between eSNPs and the expression of genes. In total, association was established between 151,571 eSNPs and 11,558 genes. SNPs located in 11,558 genes were mapped to genes as described above. Overall 3.5 × 10^6 ^eSNP-SNP pairs were generated.

### Test of interactions

The following logistic regression model as implemented in Plink was applied to test the interactions among SNPs using the option -epistasis [34]:

$$\text{logit}\left(\text{P}\right)={\text{b}}_{0}+{\text{b}}_{1}\times \text{rs}1+{\text{b}}_{2}\times \text{rs}2+{\text{b}}_{3}\times \text{rs}1\times \text{rs}2,$$

where P is the probability of being affected, b_0 _is the intercept, b_1_, b_2_, and b_3 _are coefficient terms, and rs1 and rs2 are the additive codes for the two SNPs (i.e. the number of susceptibility alleles, 0,1 or 2). The biological meaning of these terms are as follows: b_0_, the baseline odds of disease; b_1 _and b_2 _are odds of disease due to the two SNPs respectively; b_3_, the odds of disease due to interaction between two SNPs. They are calculated by traditional logistic regression analysis. Then, a two-sided test of the null hypothesis b_3 _= 0 is performed assuming the test statistic follows its asymptotic distribution. The Bonferroni method was used to correct for multiple hypothesis-testing separately for each of the gene, pathway, network, and eSNP levels. Corrected p-values < 0.05 were considered as significant. The associations of single SNPs with T2D were also compared to interaction p-values.

## Results and discussion

### Genes with SNP-SNP interactions

The initial analysis of this data by the WTCCC identified three SNPs significantly associated with T2D (rs9465871, rs4506565, and rs9939609)[1]. We found that most of the p-values for SNP pairs containing those SNPs are highly significant. It is not clear if there is a true association among those pairs because of the strong association of those three SNPs with T2D. Further analyses are needed to clarify this result, and thus those SNPs pairs are not presented in this work to focus the results on interactions that promote non-significant SNPs to significance.

Four genes, ZFAT, NDST3, C9orf3 and PPM1A, with one to three SNP pairs identified for each gene, were found to significantly associate with T2D in the WTCCC data set on the basis of an interaction term using the logistic regression model (Table 1). None of these four genes had significant associations with T2D when single SNPs were analyzed, as it is commonly accepted that the p-value significance threshold for a single SNP is 5 × 10^-7^[1]. However, in most cases at least one of the SNPs in the pairs had marginal (non-significant) p-values (around 10^-5^). In the case of multiple SNP pairs for one gene, one SNP was shared between pairs, which was the one with a nominal (but not genome-wide) significant p-value. Notably, the p-values for these interaction SNPs are improved by several orders of magnitude (p-value ranges from 10^-8 ^to 10^-11^) compared to the p-values from individual SNPs. The average distance between the SNPs pairs is 47 kb (ranging from 0.3 to 129), and based on their correlations it is likely that they locate in different LD blocks (Table 1). Two SNPs (rs1994385 and rs2389493) are located in the upstream of NDST3; all other SNPs are located in the intronic regions of the presumed corresponding genes.

**Table 1:** SNP pairs detected when testing among SNPs located in the same genes.

SNP1	p-value (SNP1)	SNP2	p-value (SNP2)	p-value (interaction)	Distance	correlation	Genes
rs1994385	3.7 × 10^-1^	rs2389493	3.3 × 10^-5^	1.3 × 10^-8^	8 k	0.63	NDST3
	
rs2389493	3.3 × 10^-5^	rs6534079	3.0 × 10^-1^	1.6 × 10^-8^	17 k	0.63	

rs6421008	4.8 × 10^-1^	rs7827545	1.4 × 10^-3^	1.5 × 10^-11^	54 k	0.07	ZFAT

rs356127	9.2 × 10^-1^	rs2406898	1.5 × 10^-5^	9.4 × 10^-9^	63 k	0.37	
	
rs7853126	6.5 × 10^-1^	rs2406898	1.5 × 10^-5^	1.2 × 10^-8^	129 k	0.37	C9orf3
	
rs2584803	8.3 × 10^-1^	rs2406898	1.5 × 10^-5^	1.2 × 10^-8^	73 k	0.37	

rs17097262	3.4 × 10^-1^	rs7154773	5.7 × 10^-5^	1.7 × 10^-10^	10 k	0.04	
	
rs7154773	5.7 × 10^-5^	rs10130695	2.3 × 10^-1^	3.5 × 10^-10^	6.9 k	0.04	PPM1A
	
rs12323784	2.0 × 10^-1^	rs7154773	5.7 × 10^-5^	6.1 × 10^-10^	338 bp	0.05	

Correlation: correlation coefficient between two SNPs

One significant SNP pair is detected for ZFAT (zinc finger and AT hook domain); while the individual SNPs have non-significant p-values, the correlation between them is less than 0.1, and the p-value for interaction is the most significant one (1.51 × 10^-11^). ZFAT is not well studied, but likely binds DNA and functions as a transcriptional regulator related to apoptosis and cell survival [41,42]. Two pairs of SNPs are detected for NDST3. This is annotated as a monomeric bifunctional enzyme, which catalyzes the N-deacetylation and N-sulfation of N-acetylglucosamine residues in heparan sulfate and heparin. C9orf3 contains three significant SNP pairs, and is likely a member of the M1 zinc amino-peptidase family based on the annotation in NCBI. The proposed encoded protein is a zinc-dependent metallopeptidase that catalyzes the removal of an amino acid from the amino terminus of a protein or peptide. There is no prior association with T2D reported for ZFAT, NDST3 or C9orf3, and thus they are novel targets for further study.

The fourth gene with significant SNP pairs is PPM1A, which is a member of the PP2C family of Ser/Thr protein phosphatases and reported to be indirectly associated with T2D [43]. PPM1A is involved in the IRS (insulin regulated signaling) pathway. Its function is to dephosphorylate and negatively regulate the activities of MAP kinases, which are important for insulin-signaling [44]. Moreover, it has been shown to inhibit the activation of p38 and JNK kinase cascades induced by environmental stresses [45]. Over-expression of this phosphatase is reported to activate the expression of the tumor suppressor gene TP53/p53, which leads to G2/M cell cycle arrest and apoptosis [46].

### Interactions detected by pathway analysis

**Figure 2:**
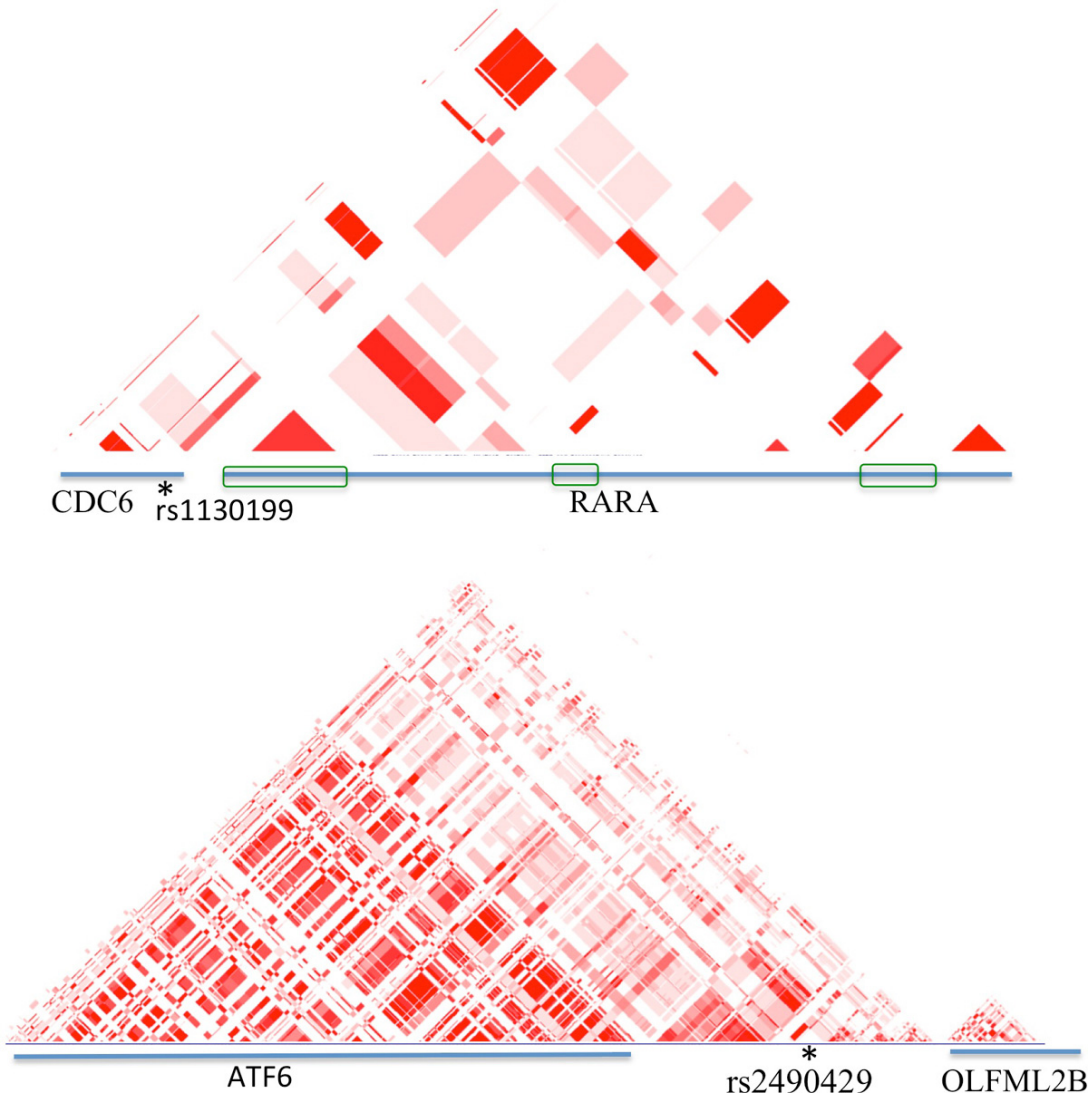
**LD blocks of two regions**. LD is calculated based on r^2 ^in the region of CDC6 and RARA, and ATF6 and OLFML2B using UCSC genome browser's HapMap LD Phased Track. The diagonal pattern illustrates the LD relationship between two regions. Blue lines represent genes. * indicates the location of the SNP. The green boxes in the upper panel represent the regions of RARA that have high association with CDC6, indicating the two genes are related. In the lower panel, although rs2490429 locates between ATF6 and OLFML2B, its neighborhood has strong association with ATF6.

The interactions among SNPs located in different genes of 655 pathways were exhaustively tested for each pathway individually. In total, 2.67 × 10^7 ^pairwise tests were performed, and the p-value cutoff for 5% significance after Bonferroni correction is 1.9 × 10^-9^. One single pair of SNPs was detected: rs1130199 and rs42537s64, with p-value 7.0 × 10^-11^, which are present in chromosomes 17 and 22, respectively (Table 2). When testing for the main effect of each SNP, both show non-significant p-values: 1.5 × 10^-5 ^(rs1130199) and the marginally significant p-values 1.3 × 10^-7 ^(rs4253764). rs4253764 is located in an intron of PPARA (peroxisome proliferator-activated receptor alpha), while rs1130199 is in an exon near the 3' region of the CDC6 (cell division cycle) gene. PPARA and CDC6 are not present in the same pathway; however, our SNP to gene assignment associated this SNP with two genes because we included 20 kb of sequence adjacent to the gene, thus including the RARA (retinoic acid receptor, alpha) gene. RARA is present in two pathways with PPARA: nuclear receptors in a lipid metabolism and toxicity pathway, and a nuclear receptor transcription pathway. Although CDC6 and RARA do not belong to a single LD block, segments of the genes have strong associations, as shown in Figure 2. Thus, the interaction may be mediated through LD with RARA.

**Table 2:** SNP pairs detected through analysis of pathway, network and eSNPs

SNP1	chr	gene	P-value (snp1)	SNP2	Chr	gene	P-value (snp2)	P-value (interaction)	Correlation	Analysis
rs1130199	17	CDC6 RARA	1.5 × 10^-5^	rs4253764	22	PPARA	1.3 × 10^-7^	7.0 × 10^-11^	0.35	Pathway

rs2490429	1	ATF6 OLFML2B	2.2 × 10^-7^	rs41433646	12	RBM19	2.2 × 10^-2^	2.9 × 10^-11^	0.15	Subnetwork

rs4253764	22	PPARA	1.3 × 10^-7^	rs41433646	12	RBM19	2.2 × 10^-2^	9.9 × 10^-10^	0.33	Subnetwork

Rs12517663	5	-	5.5 × 10^-1^	Rs3751726	16	KLHDC4	2.9 × 10^-1^	9.6 × 10^-10^	0.09	Esnp

chr stands for chromosome; Correlation: correlation coefficient between two SNPs.

PPARA is a nuclear transcription factor, which also mediates peroxisome proliferators and affects the expression of target genes involved in cell proliferation, in cell differentiation and in immune and inflammation responses. PPARA is believed to associate with diabetic "microvascular" complications (damage to the small blood vessels), and is considered as a potential treatment target for such complications [47]. Furthermore, PPARA interacts with PPARGC1A (peroxisome proliferator-activated receptor gamma, coactivator 1 alpha), which interacts with PPARG (peroxisome proliferator-activated receptor gamma), which permits the interaction of this protein with multiple transcription factors. PPARG is reported to be significantly associated with T2D in the original analysis of the WTCCC GWAS dataset [1]. The protein coded by the CDC6 gene is essential for the initiation of DNA replication, and functions as a regulator at the early steps of DNA replication. RARA regulates transcription in a ligand-dependent manner. It is implicated in the regulation of development, differentiation, apoptosis, granulopoeisis, and transcription of clock genes. Both CDC6 and RARA are less well-studied genes, and there is no T2D association with CDC6 or RARA reported so far. Our results suggest that they might be associated with T2D through their interaction with PPARA.

### Interactions detected by network analysis

**Figure 3:**
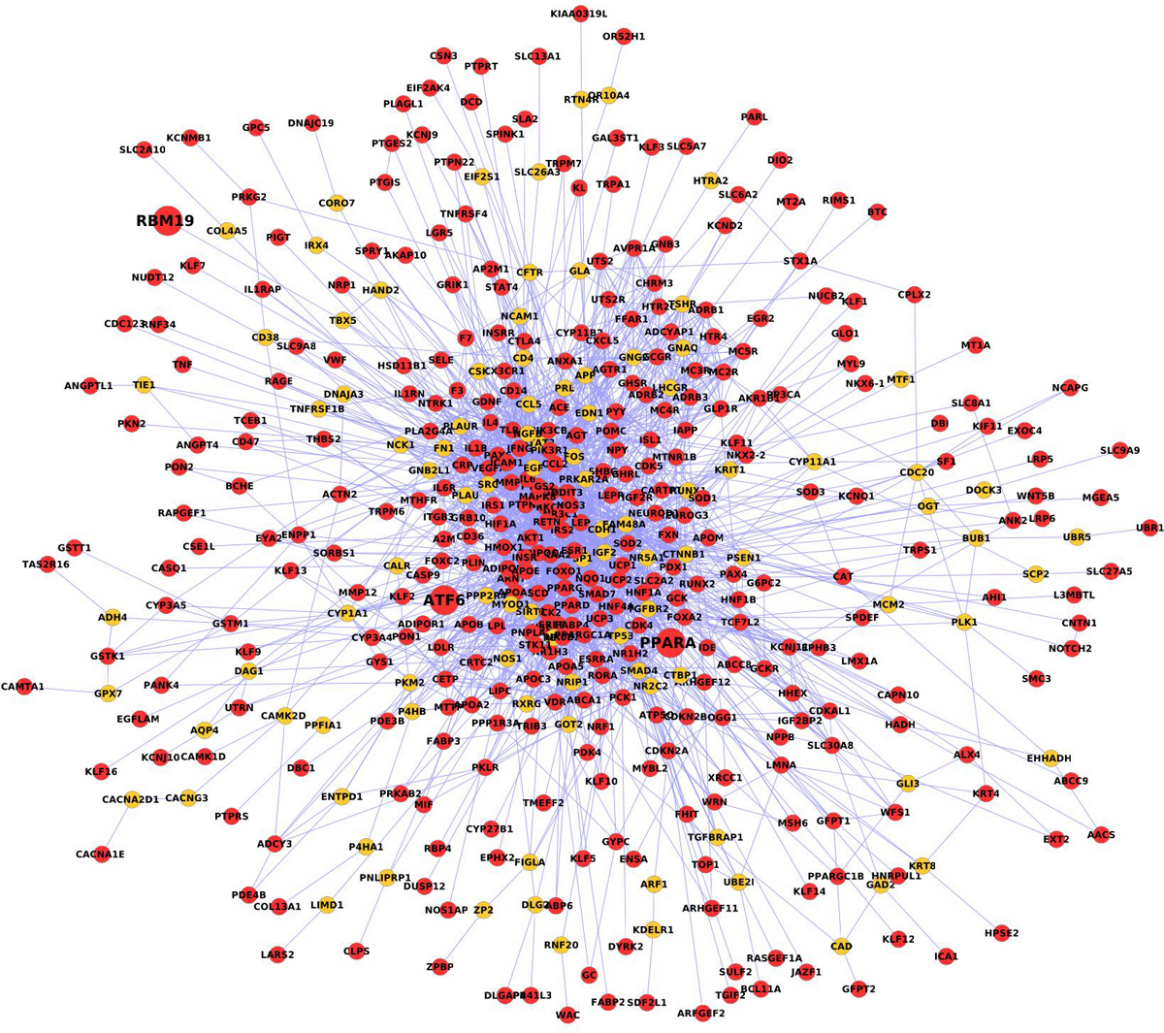
**Subnetwork associated with T2D**. The subnetwork was generated by applying the Steiner tree algorithm on a human interactome and T2D genes. Red nodes are T2D genes, the orange nodes are added to connect T2D genes using Steiner tree algorithm.

The subnetwork associated with T2D was generated by 446 T2D genes and the Steiner tree algorithm and is shown in Figure 3. The subnetwork has 453 genes with 2,374 connections. Among the initial 446 T2D genes, 354 genes have connections in the STRING database, and were retained in the subnetwork; an additional 99 genes were added to connect them. Genes in the middle of the subnetwork are highly connected: these 121 genes (25% of total genes) have 2,088 connections (85% of total connections) in the T2D network. SNPs located in all 453 genes of the subnetwork were tested for interactions exhaustively (not only the connected genes). In total, there are 4.7 × 10^7 ^SNP-SNP tests, and the cutoff p-value after correction is 1.1 × 10^-9^.

Two pairs of SNPs were found with significant p-values (Table 2), and they shared one common SNP: rs41433646, which locates in an intron of the RBM19 (RNA binding motif protein 19) gene. One of the other two SNPs (rs2490429) locates in between two genes: ATF6 (activating transcription factor 6) and OLFML2B (olfactomedin-like 2B). Although the distances between rs2490429 with ATF6 and OLFML2B are almost the same, ATF6 and rs2490429 belong to the same LD block, as illustrated in Figure 2. Thus it is likely that ATF6 and rs2490429 are associated to each other. Interestingly, the third SNP is rs4253764, which is part of the SNP pair detected in the above pathway analysis and located in the intron region of PPARA.

The protein encoded by ATF6 is a transcription factor. During endoplasmic reticulum (ER) stress, ATF6 activates target genes for the unfolded protein response. There are reports that its polymorphisms are associated with diabetes in various populations, such as Dutch Caucasians and Indians [48,49]. OLFML2B locates close to ATF6, and is a relatively less known gene. RBM19 encodes a nuclear protein that contains six RNA-binding motifs, which may be involved in regulating ribosome biogenesis. No strong association with T2D has been reported for RBM19.

**Figure 4:**
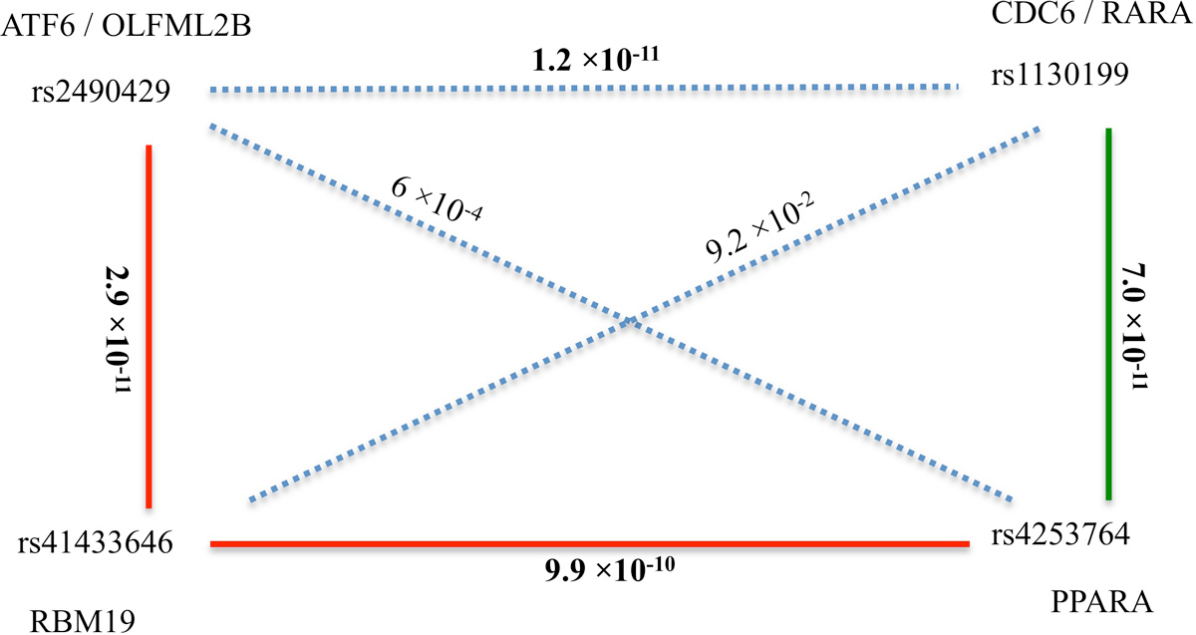
**Network constructed by interactions of SNPs detected by pathway and network analysis**. Solid lines represent interactions detected by pathway or network analysis. The red lines represent interactions detected by network analysis, while the green line represents interaction detected by pathway analysis. The pathway and network analysis led to the detection of three interactions among four SNPs. Some SNPs pairs (such as rs2490429 and rs1130199), are never present in same pathway or network, thus the associations between them are not tested in either pathway or network analysis. Additional testing of these SNP pairs was conducted. The p-values for the dotted line come from these additional analyses. The significant p-values are in bold. The genes related to SNPs are positioned close to SNPs.

The pathway and network analysis led to the detection of three interactions among four SNPs (Figure 4). One SNP (rs4253764) was detected in both analyses. rs1130199 is absent in the subnetwork of Figure 3, while rs2490429 and rs41433646 are not in the pathway set; consequently, some pairs among the four SNPs were not tested for interactions in either pathway or network analysis. Additional testing of these SNP pairs showed that the p-value for interaction of rs2490429 and rs1130199 is 1.2 × 10^-11^, and p-values for the other two pairs are non-significant. Interestingly, four out of six possible SNP pairs in Figure 4 have significant p-values, which suggests that the network containing them is strongly associated with T2D. The biological mechanism behind this network and its association with T2D are interesting subjects for further detailed investigation.

### Interaction detected for a gene and its eSNP

The above analysis focused on testing of interactions among SNPs based on gene-gene based frameworks. We also attempted to explore the interactions of SNPs through an examination of eSNPs and associated genes whose expression is altered by the eSNPs. In total, 3.5 × 10^6 ^tests were performed for the search of interaction between expression altered genes and eSNPs. One SNP pair was detected with significant p-value after correction (Table 2). rs12517663 is located in an intergenic region of chromosome 5, and significantly affects the expression of KLHDC4 (kelch domain containing 4, chromosome 16) in lymphoblastoid cell lines with p-value < 1.0 × 10^-5^. The interaction between rs12517663 and rs3751726 (located in the neighborhood of KLHDC4) is significantly associated with T2D (p-value 9.6 × 10^-10^). The function of KLHDC4 is unknown. However, Kelch proteins are commonly seen to associate with actin tails.

### Assumptions for association testing

Two major assumptions of the analysis are: 1) the numbers of individuals in the nine genotype cells of the contingency table for two SNPs, for both cases and controls, are large enough to assume an asymptotic null distribution for the test statistic, and 2) our results are not confounded by population structure. To be sure that the first assumptions does not invalidate our results we made sure that each of the pairs of SNPs showed a significant difference between cases and controls using Fisher's exact test, as implemented in the R function fisher.test. This was an overall test for the effect of the two SNPs, not just for the interaction term in our logistic regression model. The results showed the p-values for all detected SNP pairs are still significant after correction. Regarding the second assumption, we calculated the 10 top principal components using ancestry informative marker SNPs with Plink [50], and then we repeated each 2-SNP analysis where we found a significant interaction but including as covariates in the model the 10 top principal components of all the SNPs. The results showed that there is virtually no change in p-values of the coefficients of the interaction terms, which indicates that the associations detected are not caused by population stratification.

## Conclusion

In conclusion, this study presented several approaches to search for disease-associated gene-gene interactions from GWAS data based on prior biological knowledge and discrete biological frameworks. This is in stark contrast to the single locus approach and the results provide many interesting genes and interactions with significant p-values. While some of the identified SNPs are linked to genes that are well known for their association with T2D, such as PPARA, others are novel, and potentially provide new avenues for further research. The original analysis based on single locus models revealed only three genes associated with T2D: PPARG, KCNJ11 and TCF7L2 [1]. Our analysis uncovered 12 additional genes that might be associated with the disease through the statistical interaction of SNP pairs in the same or different genes. We believe that the multiple-locus models presented in this and previous studies, such as the method based on adjacent SNPs [27], may outperform the single locus model to detect true associations, where the addition of relevant biological knowledge can dramatically improve the efficiency of the search.

## List of abbreviations used

GWAS: genome wide association study; SNP: single nucleotide polymorphism; eSNP: expression single nucleotide polymorphism; eQTL: expression quantitative trait loci; T2D: type two diabetes; LD: linkage disequilibrium; WTCCC: wellcome trust case control consortium.

## Competing interests

The authors declare that they have no competing interests.

## Authors' contributions

YL designed the study, collected data, carried out the analysis and drafted the manuscript; SM programmed the pipeline; TF, XZ and RCE helped on the statistical testing; MK collected and managed data, and help on the study design; MRC conceived of the study and participated in its design and coordination. All authors have read and approved the final manuscript.
